# ^226^Ra, ^210^Pb, ^210^Bi and ^210^Po deposition and removal from surfaces and liquids

**DOI:** 10.1007/s10967-012-2180-5

**Published:** 2012-09-18

**Authors:** M. Wójcik, G. Zuzel

**Affiliations:** M. Smoluchowski Institute of Physics, Jagiellonian University, Reymonta 4, 30-059 Cracow, Poland

**Keywords:** Radium adsorption, Lead removal, Distillation, Water extraction, Etching of germanium surface

## Abstract

Deposition of ^226^Ra from water on nylon was investigated. Measurements performed for different pH and different radium concentrations in the water gave similar absolute activities deposited on the foil surface. Obtained results were used to estimate the amount of ^226^Ra plated-out on the nylon scintillator vessel in the solar neutrino experiment BOREXINO during filling of the detector. Another problem studied in the frame of BOREXINO was the removal of ^210^Pb from its organic liquid scintillator by applying distillation and water extraction. After several tests had been performed for both methods it was found that after the water extraction the initial lead content in the scintillator sample was reduced only accordingly to the ratio of the volumes of the applied liquids (simple dilution). In contrast to this, distillation was very effective providing in the best case a ^210^Pb reduction factor higher than 100. Removal efficiencies of the long-lived ^222^Rn daughters during etching from surfaces of standard and high purity germanium were investigated in the frame of the GERDA experiment, which aims to search for neutrino-less double beta decay of ^76^Ge. The standard etching procedure of Canberra used during production of high purity n-type germanium diodes was applied to germanium discs, which had been exposed earlier to a strong ^222^Rn source for its progenies deposition. In contrast to copper and stainless steel, ^210^Pb, ^210^Bi and ^210^Po was removed from germanium very efficiently. An evidence of a reverse process was also observed—the isotopes were transferred from the etchant to the clean germanium surface.

## Introduction

**Fig. 1:**
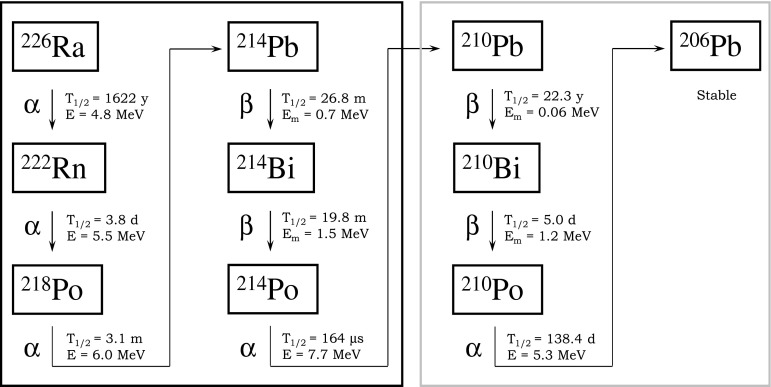
Part of the ^238^U chain starting from ^226^Ra with the most important decay products. The short- (*left*) and the long-lived (*right*) sub-chains are marked by frames

Natural radioactive isotopes form the ^238^U chain pose in many cases the most relevant background source in experiments looking for rare nuclear processes, like interactions of low-energy neutrinos, interactions of cold dark matter particles or neutrino-less double beta decay. A part of the chain starting from ^226^Ra is shown in Fig. 1. Short-lived isotopes up to ^210^Pb can be linked to radium whose activity can be measured with high sensitivity and high precision through a ^222^Rn emanation technique [1]. Radioactive equilibrium in the chain is broken very often at ^210^Pb due to its long half-live of 22 years. This isotope is usually plated-out on surfaces and may remain as a main residual contamination appearing only after some time through ^210^Bi (*T*_1/2_ = 5.0 d, *E*_max_(β) = 1.2 MeV) or ^210^Po (*T*_1/2_ = 138.4 d, *E*(α) = 5.3 MeV). ^226^Ra and ^210^Pb are therefore often the most relevant isotopes in the short- and long-lived parts of the uranium chain. Radioactive decays occurring there may contribute significantly to the background of the experiments looking for rare nuclear processes through different kinds of radiation and its wide energy range.

## Experimental

### ^226^Ra adsorption on nylon

Deposition of ^226^Ra on nylon was investigated in the frame of BOREXINO [2]. The experiment is located deep underground (3800 m of water equivalent) in the Hall C of the Laboratori Nazionali del Gran Sasso (Italy), where the muon flux is suppressed by a factor of ~10^6^ with respect to the surface. The detector was designed to register in real time the low-energy solar neutrinos through elastic neutrino–electron scattering occurring in large volume unsegmented liquid scintillator (LS). It is contained in a spherical nylon vessel (Inner Vessel, IV, made out of a 0.125 mm thick film) and observed by 2212 PMTs. Since the expected signal coming from the solar ^7^Be neutrinos (primary species to be measured in the experiment) was at the level of about 40 events/day/100 ton, ultimately low background was required. One of its sources could be ^222^Rn produced by decays of ^226^Ra deposited on the IV surface during filling of the detector with ultra-pure water with the specific ^226^Ra activity of about 1 mBq/m^3^ [3]. This water was later replaced by LS (preliminary filling with water was necessary for safety reasons). Due to the large volume of the detector target (about 280 ton) the total ^226^Ra activity contained in the water filled into the nylon vessel was high (~0.3 Bq) and therefore it was necessary to investigate what was the fraction, which could be plated-out on the IV surface. ^222^Rn produced by decays of deposited radium can diffuse into the most internal 100 ton of the scintillator (fiducial volume, FV, only neutrino interactions from this part are accounted for) increasing the overall background of the detector.

**Fig. 2:**
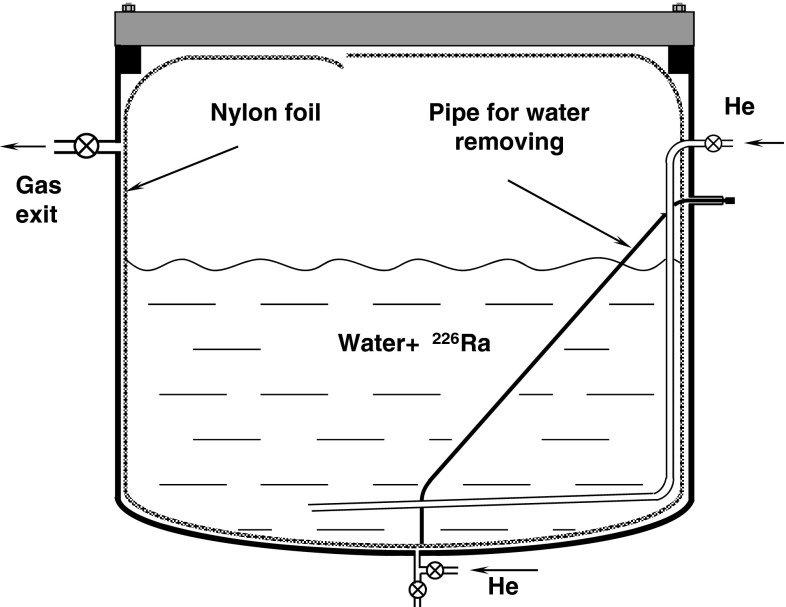
Schematic view of the apparatus used to investigate deposition of ^226^Ra on the nylon foil to be used in BOREXINO

For the ^226^Ra deposition tests an emanation chamber with a volume of 20 l was used [4]. A nylon bag was formed out of a foil (Capron) considered to be used in BOREXINO and placed inside the chamber as shown in Fig. 2. It was next filled with ultra-pure water, to which in the following step known activity of ^226^Ra (coming from a certified solution) was added. After some time when the radium could be deposited on the foil surface (between 3 weeks and 4 months for different tests) the water was pumped out from the bag through a special pipe going to its deepest point. After each step of the above procedure the total ^226^Ra activity inside the chamber was precisely measured through ^222^Rn.^1^ Radon was extracted from the vessel using pure He as a carrier gas, collected in a charcoal trap and filled into a proportional counter for registration of its alpha decays [1]. Determination of the ^226^Ra activity in each configuration allowed to precisely determine how much ^226^Ra was present in the water, and how much was adsorbed on the foil. Appropriate corrections for amounts of water removed during radon extractions (sparging with He) and left over on the foil after water removal were applied. Three series of measurements were performed for different pH values of the water: 2.8, 5.7, 6.8. Respectively, different concentrations of ^226^Ra were applied: 1.1, 2.5, 0.5 mBq/l, as well as different exposure times (3 months, 3 weeks and 4 months, respectively). The surface area of the used foils was always about 0.2 m^2^ and the fraction being in direct contact with the ^226^Ra-rich water equaled to 0.15 m^2^. All relevant values and obtained activities for all three runs are collected in Tables 1 and 2.

**Table 1:** ^226^Ra activities inside the emanation chamber measured at different stages of the tests of radium deposition on nylon. Three series of measurements were performed for different pH values and different ^226^Ra concentrations in the water. After the description of the actually investigated setup a brief information about the following step is given

Configuration → following action	Measured ^226^Ra activities (mBq)		
	pH = 2.8	pH = 5.7	pH = 6.8
Blank of the emanation chamber → foil installation	0.065 ± 0.015	0.073 ± 0.021	0.056 ± 0.015
Emanation chamber + foil → adding water (8.34 l | 7.77 l | 8.30 l)	0.10 ± 0.02	0.10 ± 0.03	0.063 ± 0.015
Emanation chamber + foil + water → adding ^226^Ra (9.1 mBq | 17.9 mBq | 4.3 mBq)	0.14 ± 0.04	0.17 ± 0.03	0.13 ± 0.02
Emanation chamber + foil + water + ^226^Ra → removing water (3 m | 3 weeks | 4 m)	9.20 ± 0.40	18.10 ± 0.80	4.30 ± 0.20
Emanation chamber + foil + rest of the water + deposited ^226^Ra → removing the foil	0.31 ± 0.05	0.35 ± 0.04	0.20 ± 0.04
Blank of the emanation chamber	0.073 ± 0.021	0.057 ± 0.018	0.064 ± 0.016

**Table 2:** Measured activities and concentrations of ^226^Ra in the water and activities of ^226^Ra deposited on the foil for all three performed tests. In the last row percentage of the amount of radium deposited on nylon in each test is given

Quantity	pH of the water		
	2.8	5.7	6.8
^226^Ra activity in the water *A*_Ra_ (mBq)	9.1 ± 0.5	18.8 ± 0.8	4.3 ± 0.2
^226^Ra concentration in the water *C*_Ra_ (mBq/l)	1.1 ± 0.1	2.3 ± 0.1	0.5 ± 0.1
^226^Ra activity on/in the foil *A*_P_ (mBq)	0.18 ± 0.07	0.15 ± 0.08	0.13 ± 0.05
Fraction of ^226^Ra adsorbed on/in the foil *P* (%)	2.0 ± 0.8	0.8 ± 0.5	3.0 ± 1.2

### Removal of ^210^Pb from the BOREXINO liquid scintillator

Besides ^226^Ra/^222^Rn, ^210^Pb was one of the major concern in the BOREXINO experiment. If present in the liquid scintillator (e.g. as a result of an exposure to air-born ^222^Rn) it would produce ^210^Bi, which due to relatively short half-life will reach equilibrium with lead after only 2 weeks. Beta decays of ^210^Bi (*E*_max_(β) = 1.2 MeV) will then directly disturb observations of neutrinos through the scattered electrons registered for energies above 250 keV. It was therefore necessary to find an effective method of ^210^Pb removal, which can be applied in the scintillator purification process taking place just before filling of the detector. LS used by BOREXINO consists of pseudocumene solvent (PC, 1,2,4-trimethylbenzene, C_6_H_3_(CH_3_)_3_, ρ = 0.87 g/cm^3^) and the fluor (PPO, 2,5-diphenyloxazole, C_15_H_11_NO) at a concentration of 1.5 g(PPO)/l(PC). Distillation and water extraction were considered (for pure PC^2^) as the most suitable purification processes for the experiment (also considering other radioactive isotopes). Both were tested by us in the laboratory scale using PC artificially contaminated with ^210^Pb through a long (a few months) exposure to a ^222^Rn-rich gas. Decay rates of ^210^Pb before and after processing the samples were determined by observing the 46.5 keV gamma line with an *n*-type high purity germanium detector (HPGe, 25 % relative detection efficiency, aluminum window) [5].

**Fig. 3:**
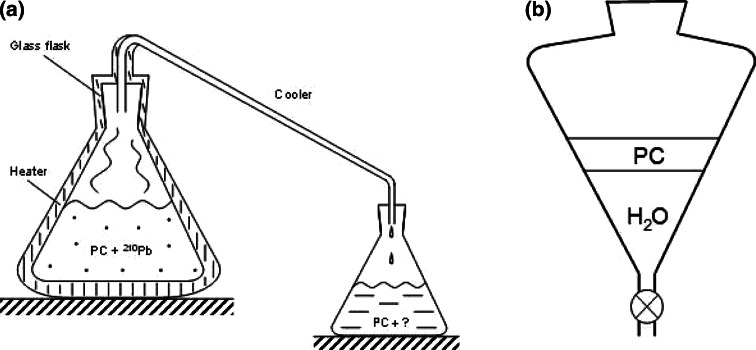
Schematic views of the setups used for the tests of ^210^Pb removal from the BOREXINO pseudocumene through its distillation (**a)** and water extraction (**b**)

PC distillation efficiency was investigated by using a simple system shown in Fig. 3a. The flask containing the polluted sample (~200 ml, its ^210^Pb content was determined in a 35 cm^3^ sample just before the test) was heated over its entire surface. Distilled liquid was collected in a clean bottle and its small fraction (35 cm^3^) was screened for the ^210^Pb content. Two tests were performed, for which the initial lead concentrations in PC differed by one order of magnitude. The obtained results are collected in Table 3.

**Table 3:** Results of the PC distillation tests with respect to ^210^Pb removal. Volumes of the measured samples were always 35 cm^3^

Activity of ^210^Pb in PC before distillation (cpm)	Activity of ^210^Pb in PC after distillation (cpm)	^210^Pb reduction factor
0.045 ± 0.005	<0.007 (90 % C.L.)	>6
0.425 ± 0.013	<0.004 (90 % C.L.)	>106

Water extraction is in general a purification technique reducing impurities in organic liquids. The availability of ultra-pure water by deionization and distillation makes it an easily applicable method. As most impurities are of polar nature, they prefer to dissolve in polar liquids like water. As soon as it comes in contact with nonpolar liquid scintillator, a big part of polar scintillator contaminants (like ^210^Pb) dissolve in water. The efficiency of this process improves with a higher contact time and a better mixing quality between the two liquid phases. The equilibrium to be reached depends on the impurity solubilities in both liquids, varying with different pH values.

In our tests we used again PC loaded with ^210^Pb and distilled water (pH = 6.0). Their volumes were the same and they were mixed in a glass vessel shown schematically in Fig. 3b. Four runs were performed for different times, which elapsed between the end of mixing (extraction) and the moment when the vessel was drained and both liquids were refilled into separate containers for analysis of their ^210^Pb content. The obtained results are collected in Table 4. Amounts of the investigated samples were always the same (35 cm^3^).

**Table 4:** Results of the PC water extraction tests with respect to ^210^Pb removal. Volumes of the measured samples were always 35 cm^3^

Activity of ^210^Pb after water extraction (cpm)		Time elapsed since the end of mixing	Separation coefficient *S*
In water	In PC		
0.103 ± 0.006	0.021 ± 0.003	5 days	5.0 ± 0.6
0.106 ± 0.005	0.021 ± 0.002	1 day	5.0 ± 0.8
0.068 ± 0.006	0.025 ± 0.004	Few hours	2.7 ± 0.7
0.024 ± 0.003	0.028 ± 0.004	Immediate separation	0.9 ± 0.2

### Removal of ^222^Rn daughters from germanium surfaces

The problem of surface contamination with the long-lived daughters of ^222^Rn is of great interest for many experiments looking for rare nuclear processes. One of the examples is the GERDA (Germanium Detector Array) project [6], where high purity germanium (HPGe) crystals made out of material enriched with ^76^Ge will be used to search for the neutrino-less double beta decay. Due to the expected very low background of the detectors, the crystal’s surface contamination may constitute one of the most critical residual radioactivity sources.

In the frame of GERDA several tests of ^210^Pb, ^210^Bi and ^210^Po removal and deposition during etching of normal purity germanium (NPGe, so called “optical”, in our case its surface was mechanically polished) and high purity germanium (HPGe with the surface finish equivalent to the produced high purity Ge diodes) samples were performed. To investigate the removal effectiveness the samples were artificially contaminated by exposing them for some months to a strong radon source. They were prepared as disc of 50 mm in diameter and 3 mm thick. Usually each side of the disc was screened separately before and after treatment.

For the investigation of deposition efficiencies (transfer of isotopes from the etchant to the surface) of lead, bismuth and polonium on a clean high purity germanium surface the water to be used for preparation of the etching solution was contaminated by exposing it to a ^222^Rn-rich gas.

The germanium samples were processed by Canberra according to their standard etching procedure (using CP4 solution) applied during production of HPGe detectors. Details of this work can be found in [7]. Here only the main achievements are summarized in Table 5 (etching of a contaminated NPGe sample), Table 6 (etching of a contaminated HPGe sample) and Table 7 (etching of a clean HPGe sample in the contaminated CP4 solution).

**Table 5:** Results of etching of the NPGe sample. The averaged reduction factor is calculated taking into account values (or limits) obtained for both disc sides. ^210^Pb on side “b” was not measured

Isotope	Disc side	Initial count rate (cpm)	Count rate after cleaning (cpm)	Reduction factor *R*	Averaged reduction factor *R*_av_
^210^Pb	a	2.08 ± 0.11	<0.02	>104	>104
	b	3.43 ± 0.23	–	–	
^210^Bi	a	42.7 ± 1.3	<0.18	>237	>427
	b	67.9 ± 2.0	<0.11	>617	
^210^Po	a	42.4 ± 1.3	0.04 ± 0.01	1060 ± 267	2,300
	b	71.7 ± 1.9	0.02 ± 0.01	3585 ± 1795

**Table 6:** Reduction of ^210^Pb, ^210^Bi and ^210^Po count rates on the high purity germanium disc. Only one side of the disc was investigated

Isotope	Initial count rate (cpm)	Count rate after etching (cpm)	Reduction factor *R*
^210^Pb	0.717 ± 0.011	<0.001	>717
^210^Bi	14.70 ± 0.12	0.017 ± 0.009	865 ± 458
^210^Po	11.88 ± 0.19	0.102 ± 0.006	117 ± 7

**Table 7:** Count rates of ^210^Pb, ^210^Bi and ^210^Po on the clean high purity germanium disc etched in a solution made with contaminated water. Only one side of the disc was investigated. The second column represents the blank values (clean HPGe disc). The third column shows the directly measured count rates after etching, their background-corrected equivalents are given in the fourth column

Isotope	Initial count rate (cpm)	Count rate after etching (cpm)	Count rate increase (cpm)	Count rate increase factor *B*_R_
^210^Pb	0.0163 ± 0.0009	0.023 ± 0.001	0.0066 ± 0.0013	1.4
^210^Bi	0.111 ± 0.006	0.217 ± 0.007	0.106 ± 0.009	1.9
^210^Po	0.064 ± 0.005	0.087 ± 0.006	0.023 ± 0.007	1.4

## Results and discussion

An alpha, a beta and a gamma spectrometer was used to register ^210^Po, ^210^Bi and ^210^Pb decays, respectively. For all the measurements related to purification and surface cleaning tests we were interested only in relative count rates (before and after processing of the samples). Therefore, none of the used detectors had to be calibrated. However the same detector–sample geometry had to be always preserved. The quoted results uncertainties include in most cases only statistical errors and are given as one standard deviation. Upper limits are calculated for 90 % confidence level.

### ^226^Ra adsorption on nylon

As appears from Table 2 the activity of ^226^Ra deposited on the foil was for all three runs very similar and independent on pH (in contrast to what has been observed e.g. for glass [8]), as well as on the initial radium concentration in the water. It looks like the surface was saturated or the radium was penetrating the bulk of the material together with water (it is known that the foil soaks the water increasing its own mass up to 10 %) and was later “visible” through emanated ^222^Rn [9].

The quoted in Table 2 values of the ^226^Ra activity in the water (*A*_Ra_), ^226^Ra concentration in the water (*C*_Ra_), ^226^Ra activity deposited on the foil (*A*_P_) and the fraction of ^226^Ra adsorbed on/in the foil (*P*) were obtained by taking into account appropriate results from the following measurement steps as listed in Table 1 and the necessary corrections for losses of water due to the applied measurement procedure (humidifying the He gas used to extract ^222^Rn, rests remained on the foil after removing the water from the emanation chamber). In all runs it was about 0.5 % of the initial water volume filled into the chamber. The quoted uncertainties of *A*_P_ are relatively large (from 40 to 50 %) because in many cases the measured activities were very close to the detection limit of the used apparatus. Since most of the calculations were based on subtractions the relative errors increased. The fraction *P* of ^226^Ra deposited/adsorbed on the nylon varied between 1 and 3 % and essentially followed the initial radium concentration in the water (*A*_P_ ~ constant).

For the estimation of the expected background in the BOREXINO detector caused by ^226^Ra adsorbed from the water on the IV surface an appropriate scaling of the obtained laboratory results was needed. For this purpose it was assumed that the ratio of the ^226^Ra volume (in the water) to the surface (on the foil) concentration is constant at given conditions: *C*_SF_/*C*_Ra_ = constant. The water used in BOREXINO has pH = 7.0 and its radium specific activity is 1 mBq/m^3^. Since there is practically no dependence of *A*_P_ on pH for calculations of *C*_SF_ we can average the *A*_P_ values over all performed measurements what gives 0.15 mBq, thus *C*_SF_ ≈ 1 mBq/m^2^ (the foil surface area being directly in contact with the water was ~0.15 m^2^) and *C*_SF_/*C*_Ra_ = 2000/m (*C*_Ra_ = 0.5 mBq/l as for the test with pH = 6.8). Scaling the results to the BOREXINO IV (surface area *S*_IV_ = 227 m^2^) one gets the total activity of (*A*_SF_)_BX_ = 380 μBq. Assuming that ^222^Rn produced from this radium will be homogeneously distributed in the entire LS volume of 280 ton (the worst case scenario), its activity expected in the Fiducial Volume should be about 11 dpd (decays per day). This is still acceptable (40 events/day is expected form the ^7^Be neutrinos) because majority of these events may be still recognized and rejected by registration of the Bi-Po coincidences and by pulse shape analysis (identification of alpha decays). This finding could be also cross-checked with the results obtained with the Counting Test Facility (CTF) [10], a prototype of the BOREXINO detector, where the scintillator vessel had 1 m radius. Repeating the above calculations one gets here the expected radon signal of (*A*_SF_)_CTF_ = 23 μBq = 2 dpd, which was consistent with the measured value.

### Removal of ^210^Pb from the BOREXINO liquid scintillator

The results collected in Table 3 show that the distillation was very effective in removal of ^210^Pb from the BOREXINO liquid scintillator solvent and it was applied as a main purification process during the detector filling. In our laboratory tests the best achieved activity reduction was better than 100. It was also observed that all the ^222^Rn present initially in PC was transferred completely to the distilled fraction (measurable through gamma lines of ^214^Bi).

In contrast to distillation the water extraction was rather inefficient (see Table 4). The highest value of the separation factor *S* (*S* = *N*_w_/*N*_PC_, where *N*_w_ and *N*_PC_ are the count rates registered for the water and the PC samples after extraction) was ~5. It was obtained when the liquids after mixing were kept in the vessel for 5 days (or 1 day). If this time was shorter, a few hours only, *S* was also considerably smaller (*S* ≈ 3). In case the water and PC were separated and taken for the analysis immediately after they had been mixed, *S* was only about 1. These results may be explained if ^210^Pb after the water extraction was distributed between both liquids accordingly to their volumes (simple dilution). Since in our case the amounts of the water and PC were always the same the last test gave *S* ≈ 1. In other cases the liquids stayed for some time after mixing/separation in the vessel and probably lead was transferred to the water (which due to higher density was always on the bottom) simply by sedimentation (typical for solid states), which proceeded within a day scale.

Inefficiency of the water extraction was also confirmed later directly by BOREXINO when the scintillator was processed in this way and practically no influence on its ^210^Pb/^210^Bi content was noticed.

### Removal of ^222^Rn daughters from germanium surfaces

Etching of germanium removes very efficiently all three long-lived radon daughters from its surface. It seems however, that the surface finish and/or material properties have an impact on the obtained activity reduction factors. For the normal quality germanium with mechanically polished surface (see Table 5) higher removal efficiencies were obtained for ^210^Po (in the range of 1000) than for ^210^Pb and ^210^Bi (limits of a few hundred). The HPGe disc with the standard Canberra surface finish (see Table 6) showed a slightly different trend; ^210^Po was reduced by two orders of magnitude while ^210^Bi and ^210^Pb by almost three orders of magnitude.

Considerable amounts of ^210^Pb, ^210^Bi and ^210^Po were transferred from the artificially polluted CP4 solution to the clean HPGe surface. This is an experimental fact shown by a direct measurement—their registered count rates increased up to 100 % (with respect to the background rates; see Table 7) after processing the disc in the contaminated etchant. Their absolute transfer efficiencies were roughly estimated to be at least (only limits are available) in the range of 0.2–1.2 % [7]. This finding may be important since the isotopes, especially ^210^Pb, are long-lived and their presence on the HPGe crystals in future ton-scale neutrino-less double beta decay experiments cannot be tolerated.

## Conclusions

Investigations of ^226^Ra deposition on the Capron nylon foil showed that during the initial filling of BOREXINO with water the amount of radium deposited on the IV surface will not influence significantly the background of the detector. In the worst case, assuming that ^222^Rn produced by decays of the plated-out ^226^Ra will be homogeneously distributed in the entire LS volume, it will produce about 11 dpd in the Fiducial Volume, which is still acceptable. In the case we assume that radon can move in LS only due to its diffusion, the expected signal (in FV) would be completely negligible. Since the radon diffusion length in PC is relatively short (about 2 cm) [9] practically all atoms would decay close to their origin, which is the surface of the Inner Vessel. The radon distribution in the target mass determined during the operations of the CTF and later also of the BOREXINO detector showed that in reality the liquid scintillator is slightly mixed. This is probably due to slow convection motions resulting in a kind of effective radon diffusion, which is about 10 times higher compared to the measured value [9].

The tests of PC purification showed that distillation is very effective in ^210^Pb removal. This also indicates that lead does not form volatile compounds with hydrocarbons. Water extraction on the other hand gave only a simple dilution of lead.

We also found out that the standard etching procedure applied by Canberra in production of the HPGe diodes removes very effectively all the three long-lived ^222^Rn daughters from the high- and normal purity germanium surfaces. These results can be compared to the corresponding ones obtained for copper and stainless steel and reported in [5]. The main conclusion is here that the best reduction factors were achieved for germanium where ^210^Pb, ^210^ Bi and ^210^Po activities on its surface were reduced by two to three orders of magnitude [7]. The lowest removal efficiencies have been observed for copper, where polonium was practically not affected at all. Stainless steel is somehow in-between.

## Footnotes

1 This concerns also the blank value of the empty emanation chamber and the activity of added ^226^Ra (although it was known).

2 Pseudocumene is a solvent for the liquid scintillator therefore both terms are used interchangeably.
